# Identification of *Hippophae* species (Shaji) through DNA barcodes

**DOI:** 10.1186/s13020-015-0062-9

**Published:** 2015-10-13

**Authors:** Yue Liu, Wei Sun, Chuan Liu, Yaqin Zhang, Yilong Chen, Ming Song, Gang Fan, Xia Liu, Li Xiang, Yi Zhang

**Affiliations:** College of Ethnic Medicine, Chengdu University of Traditional Chinese Medicine, Chengdu, 611137 China; Institute of Chinese Materia Medica, China Academy of Chinese Medical Sciences, Beijing, 100700 China; School of Chemical Engineering, Wuhan University of Technology, Wuhan, 430070 China

## Abstract

**Background:**

The morphological identification of different *Hippophae* species (Shaji) was difficult. This study aims to discriminate between medicinal and non-medicinal *Hippophae* species by DNA barcodes, the ITS2, *psbA*-*trnH*, and a combination of ITS2 and *psbA*-*trnH* (ITS2 + *psbA*-*trnH*).

**Methods:**

DNA was extracted from the dried fruit samples. Primer pairs ITS2F/3R for ITS2 and *psbA*F/*trnH*R for *psbA*-*trnH* were used for PCR amplification. The purified PCR products were bidirectionally sequenced. Genetic distances were calculated according to the Kimura 2 parameter model and phylogenetic tree was constructed based on neighbor-joining (NJ) method, barcoding gap was also analyzed to assess identification efficiency.

**Results:**

Amplification and sequencing efficiencies for both ITS2 and *psbA*-*trnH* were 100 %. Sequence data revealed that ITS2 + *psbA*-*trnH* was the most suitable candidate barcode at the species and subspecies level. The closely related *Hippophae* species were effectively differentiated in the NJ tree.

**Conclusion:**

The combination of the two loci, ITS2 + *psbA*-*trnH* is applicable to the identification of medicinal and non-medicinal *Hippophae* species.

## Background

In *Hippophae* (Fam. Elaeagnaceae) (Shaji), seven species and 11 subspecies have been identified worldwide [1, 2]. In China, there are seven species and seven subspecies of *Hippophae*, which are mainly distributed from the Hengduan Mountains to the Qinghai-Tibet Plateau [3–6].

Both the fruits and leaves of *Hippophae* species possess abundant nutritional properties and bioactive compounds [7–9], *i.e.*, high level of vitamin C [10, 11]. *Hippophae* species have been widely used in food, pharmaceutical, and health care products [12, 13].

Medicinal *Hippophae* species are used in Chinese medicine (CM) and Tibetan medicine for their antioxidant and anti-tumor activities, to improve lipid metabolism and enhance immunity [14, 15]. The dried fruits are used as remedies for cardiovascular disease; liver, stomach, and spleen disorders; as well as lung and throat phlegm [14–18]. *Hippophae* species are sometimes misidentified because of the similarities in vegetative morphology [2, 5]. Furthermore, the fruits of different species are labeled with the same name and mainly sold or used in the dried form or as powders. Therefore, different species cannot be identified by only morphological characteristics and accurate identification methods are needed.

With the advantages of high PCR amplification efficiencies, DNA sequencing success rates, and discrimination power, DNA barcoding has become popular with taxonomists and has gained wide acceptance as a standard and effective method in biodiversity research and conservation genetics. It can be applied without the limitation of the samples development stages, parts and gathering time, compared with the conventional identification method [19, 20]. The Consortium for the Barcode of Life (CBOL) Plant Working Group initially recommended the coding plastid regions *rbcL* and *matK* as core barcodes for plant species [21]. However, two barcodes are not precise enough because of the low identification rate [22, 23]. The *psbA*-*trnH*, ITS, and ITS2 were subsequently suggested [23–25]. Additionally, the amplification efficiency of ITS is lower than that of ITS2, because of the multiple functional copies exist in many taxa [26]. Consequently, more than 6600 plant samples that belong to 4800 species from 753 distinct genera have been barcoded by ITS2, with 92.7 % success at the species level [23, 26–34]. The *psbA*-*trnH* intergenic spacer region from plastid DNA has also been recommended as a complementary barcode to ITS2 for a broad series of plant taxa [35].

This study aims to discriminate between medicinal and non-medicinal *Hippophae* species by DNA barcodes, using the ITS2 and *psbA*-*trnH* regions as candidate barcodes.

## Methods

### Materials

Seventy-five samples (Table 1) representing seven species and seven subspecies were collected from the major distribution areas, including Sichuan, Qianghai, Tibet, Yunnan, Beijing, and Xinjiang (China), between May and November 2013. The native wild samples were identified based on morphological features by Professor Zhang Yi referred to previous *Hippophae* research [4, 5]. Voucher specimens were deposited in the College of Ethnic Medicine, Chengdu University of Traditional Chinese Medicine. All of the ITS2 and *psbA*-*trnH* sequences were submitted to GenBank.

**Table 1:** *Hippophae* samples for testing potential barcodes

Scientific name	Haplotype		Voucher no.	Location	GenBank no.	
	ITS2	*psbA*-*trnH*			ITS2	*psbA*-*trnH*
*H. rhamnoides* subsp. *sinensis*	A1	M1	YC0546MT01	Wanlin, Jinchuan, Sichuan, China	KJ843997	KJ854997
	A2	M2	YC0546MT02	Maierma, Aba, Sichuan, China	KJ843998	KJ854998
	A2	M1	YC0546MT03	Shili, Songpan, Sichuan,China	KJ843999	KJ854999
	A2	M1	YC0546MT04	Rongrida, Rangtang, Sichuan, China	KJ844000	KJ855041
	A1	M3	YC0546MT05	Nanmenxia, Huzhu, Qinghai, China	KJ844001	KJ855000
	A1	M3	YC0546MT06	Puxi, Lixian, Sichuan, China	KJ844002	KJ855001
	A1	M3	YC0546MT07	Puxi, Lixian, Sichuan, China	KJ844003	KJ855002
	A2	M4	YC0546MT08	Chaka, Wulan, Qianghai, China	KJ844004	KJ855003
	A2	M5	YC0546MT09	Gatuo, Mangkang, Tibet, China	KJ844005	KJ855004
	A2	M1	YC0546MT10	Aba, Aba, Sichuan, China	KJ844006	KJ855005
	A2	M1	YC0546MT11	Luoerda, Aba, Sichuan, China	KJ844007	KJ855006
	A2	M3	YC0546MT12	Kehe, Aba, Sichuan, China	KJ844008	KJ855007
	A2	M3	YC0546MT13	Nawu, Hezuo, Gansu, China	KJ844009	KJ855008
	A1	M6	YC0546MT14	Laya, Kangding, Sichuan, China	KJ844010	KJ855009
	A2	M1	YC0546MT15	Chuanzhusi, Songpan, Sichuan, China	KJ844011	KJ855010
	A1	M1	YC0546MT16	Rilong, Xiaojin, Sichuan, China	KJ844012	KJ855011
	A1	M1	YC0546MT17	Fubian, Xiaojin, Sichuan, China	KJ844013	KJ855012
	A1	M1	YC0546MT18	Dawei, Xiaojin, Sichuan, China	KJ844014	KJ855013
	A2	M7	YC0546MT19	Baihuashan, Beijing, China	KM047400	KM047406
	A2	M7	YC0546MT20	Baihuashan, Beijing, China	KM047401	KM047407
	A2	M7	YC0546MT21	Baihuashan, Beijing, China	KM047402	KM047408
	A2	M7	YC0333MT09	Beijing, China	KM047403	KM047409
	A2	M7	YC0333MT10	Beijing, China	KM047404	KM047410
	A2	M2	FDC112^a^	National Institute for Food and Drug Control, China	KM047405	KM047411
*H. rhamnoides* subsp. *mongolica*	B1	N1	YC0547MT01	Buerjin, Altay, Xinjiang, China	KJ843986	KJ855021
	B1	N1	YC0547MT02	Buerjin, Altay, Xinjiang, China	KJ843987	KJ855022
	B1	N1	YC0547MT03	Buerjin, Altay, Xinjiang, China	KJ843988	KJ855023
*H. rhamnoides* subsp. *yunnanensis*	C1	O1	YC0548MT01	Gu, Bomi, Tibet, China	KJ817423	KJ854989
	C1	O1	YC0548MT02	Rewa, Milin, Tibet, China	KJ817424	KJ854990
	C1	O1	YC0548MT03	Rewa, Milin, Tibet, China	KJ817425	KJ854991
	C2	O1	YC0548MT04	Jiantang, Shangri-La, Yunnan, China	KJ939408	KJ939410
	C2	O1	YC0548MT05	Jiantang, Shangri-La, Yunnan, China	KJ939409	KJ939411
*H. rhamnoides* subsp*. turkestanica*	D1	P1	YC0549MT01	Aotebeixi, Wushi, Xinjiang, China	KJ844038	KJ855017
	D1	P1	YC0549MT02	Aotebeixi, Wushi, Xinjiang, China	KJ844039	KJ855018
	D1	P2	YC0549MT03	Tucheng, Zhada, Tibet, China	KJ844040	KJ855019
	D1	P2	YC0549MT04	Tucheng, Zhada, Tibet, China	KJ844041	KJ855020
*H. rhamnoides* subsp. *wolongensis*	E1	R1	YC0550MT01	Taiping, Maoxian, Sichuan, China	KJ844024	KJ855038
	E1	R1	YC0550MT02	Taiping, Maoxian, Sichuan, China	KJ844025	KJ855039
	E1	R1	YC0550MT03	Taiping, Maoxian, Sichuan, China	KJ844026	KJ855040
*H. rhamnoides* subsp*. caucasia*	DLA1	–	–	GenBank	JQ663574	–
	DLA1	–	–	GenBank	JQ663578	–
	DLA1	–	–	GenBank	JQ663579	–
	DLA1	–	–	GenBank	JQ663580	–
*H. rhamnoides* subsp. *rhamnoide*	DLB1	–	–	GenBank	AF440242	–
	DLB2	–	–	GenBank	JQ663575	–
*H. rhamnoides* subsp. *carpatica*	DLC1	–	–	GenBank	AF440245	–
	DLC2	–	–	GenBank	JQ663576	–
	DLC2	–	–	GenBank	JQ663577	–
*H. rhamnoides* subsp*. fluviatilis*	DLD1	–	–	GenBank	AF440248	–
	DLD2	–	–	GenBank	JQ289287	–
*H. goniocarpa*	F1	S1	YC0551MT01	Galitai, Songpan, Sichuan, China	KJ844018	KJ855027
	F1	S1	YC0551MT02	Galitai, Songpan, Sichuan, China	KJ844019	KJ855028
	F1	S1	YC0551MT03	Galitai, Songpan, Sichuan, China	KJ844020	KJ855029
*H. litangensis*	G1	T1	YC0552MT01	Jiawa, Litang, Sichuan, China	KJ844015	KJ854986
	G1	T1	YC0552MT02	Jiawa, Litang, Sichuan, China	KJ844016	KJ854987
	G1	T1	YC0552MT03	Jiawa, Litang, Sichuan, China	KJ844017	KJ854988
*H. neurocarpa* subsp. *neurocarpa*	H1	U1	YC0553MT01	Babao, Qilian, Qinghai, China	KJ844042	KJ854992
	H2	U2	YC0553MT02	Jiawa, Litang, Sichuan, China	KJ844043	KJ854993
	H2	U2	YC0553MT03	Jiawa, Litang, Sichuan, China	KJ844044	KJ854994
	H2	U1	YC0553MT04	Chali, Aba, Sichuan, China	KJ844045	KJ854995
	H1	U1	YC0553MT05	Maierma, Aba, Sichuan, China	KJ844046	KJ854996
*H. neurocarpa* subsp. *stellatopilosa*	I1	V1	YC0554MT01	Gaocheng, Litang, Sichuan, China	KJ844027	KJ855024
	I1	V1	YC0554MT02	Gaocheng, Litang, Sichuan, China	KJ844028	KJ855025
	I1	V1	YC0554MT03	Gaocheng, Litang, Sichuan, China	KJ844029	KJ855026
*H. salicifolia*	J1	W1	YC0653MT01	Lebu, Nacuo, Tibet, China	KJ844021	KJ855014
	J1	W1	YC0653MT02	Lebu, Nacuo, Tibet, China	KJ844022	KJ855015
	J1	W1	YC0653MT03	Lebu, Nacuo, Tibet, China	KJ844023	KJ855016
*H. gyantsensis*	K1	X1	YC0654MT01	Qiangna, Milin, Tibet, China	KJ843989	KJ855030
	K1	X1	YC0654MT02	Jieba, Naidong, Tibet, China	KJ843990	KJ855031
	K1	X1	YC0654MT03	Ridang, Longzi, Tibet, China	KJ843991	KJ855032
	K1	X1	YC0654MT04	Gangdui, Gongga, Tibet, China	KJ843992	KJ855033
	K2	X1	YC0654MT05	Pozhang, Naidong, Tibet, China	KJ843993	KJ855034
	K1	X2	YC0654MT06	Jiaxing, Gongbujiangda, Tibet, China	KJ843994	KJ855035
	K1	X1	YC0654MT07	Mozhugongka, Mozhugongka, Tibet, China	KJ843995	KJ855036
	K2	X1	YC0654MT08	Jiubu, Linzhi, Tibet, China	KJ843996	KJ855037
*H. tibetana*	L1	Y1	YC0655MT01	Langkazi, Langkazi, Tibet, China	KJ844030	KJ854976
	L2	Y1	YC0655MT02	Duoma, Ruoergai, Sichuan, China	KJ844031	KJ854977
	L1	Y1	YC0655MT03	Tangke, Ruoergai, Sichuan, China	KJ844032	KJ854978
	L2	Y1	YC0655MT04	Riduo, Mozhugongka, Tibet, China	KJ844033	KJ854979
	L1	Y1	YC0655MT05	Jiangrong, Hongyuan, Sichuan, China	KJ844034	KJ854980
	L1	Y2	YC0655MT06	Maiwa, Hongyuan, Sichuan, China	KJ844035	KJ854981
	L1	Y1	YC0655MT07	Nanmenxia, Huzhu, Qinghai, China	KJ844036	KJ854982
	L1	Y1	YC0655MT08	Tawa, Ruoergai, Sichuan, China	KJ844037	KJ854983
	L1	Y3	YC0655MT09	Chali, Aba, Sichuan, China	KJ855042	KJ854984
	L1	Y2	YC0655MT10	Maiwa, Hongyuan, Sichuan, China	KJ855043	KJ854985
	L1	Y1	YC0655MT11	Keledong, Dege, Sichuan, China	KJ855044	KJ854975
*E. angustifolia*	DLE1	–	–	GenBank	AF440256	–
*E. pungens*	–	DLDF1	–	GenBank	–	GQ435025

–: not acquired in this study

^a^FDC112: a reference crude drug that was purchased from National Institute for Food and Drug Control

Additional sequences belonging to four subspecies of *H. rhamnoides* which are only found in Europe were obtained from GenBank. In addition, *Elaeagnus angustifolia* and *E. pungens* sequences were downloaded from GenBank for use as outgroups in this study.

### DNA extraction, PCR amplification, and sequencing

Total genomic DNA was extracted from 50 mg of fruit dried in silica gel. DNA extractions were performed by a Plant Genomic DNA Kit (Tiangen Biotech Co., Beijing, China). Plant material was ground for 2 min at 50 Hz by a DNA Extraction Grinder (Xinzhi Biotech Co., Ningbo, China) as previously described [36]. Primer pairs ITS2F (5′-ATGCGATACTTGGTGTGAAT-3′)/ITS3R (5′-GACGCTTCTCCAGACTACAAT-3′) for ITS2 and *psbA*F (5′-GTTATGCATGAACGTAATGCTC-3′)/*trnH*R (5′-CGCGCATGGTGGATTCACAATCC-3′) for *psbA*-*trnH* were used for PCR amplification. PCRs were performed in a 25-μL volume, containing 2–3 μL of genomic DNA, 12.5 μL of 2 × EasyTaq PCR MasterMix (Aidlab Biotechnologies Co., Ltd., Beijing, China), 1.0 μL of each primer, and the total volume was adjusted to 25 µL with sterile deionized water. The reaction conditions used were the same as described previously [21, 37]. The PCR products were visualized on agarose gels (the electrophoresis was run in 1 × TBE for 20 min at a constant voltage 120 V). After electrophoresis, purified PCR products were bidirectionally sequenced by the same primers that were used for PCR in a 3730XL sequencer (Applied Biosystems, Foster, CA, USA).

### Data analysis

Proofreading and contig assembly of sequencing peak diagrams were performed by CodonCode Aligner 3.7.1 (CodonCode Co., Centreville, MA, USA). The ITS2 region was obtained by the HMMer annotation method based on the Hidden Markov model to remove the 5.8S and 28S sections at both ends of the sequences [38–40]. The *psbA*-*trnH* intergenic spacer boundary was determined according to the annotation of similar sequences in GenBank. All sequences were aligned (MUSCLE option) by MEGA 6.0 (Center for Evolutionary Medicine and Informatics, Tempe, AZ, USA) [41], and the genetic distances were calculated according to the Kimura 2 parameter (K2P) model. The distribution of intra- *vs.* inter-specific variability was assessed by DNA barcoding gaps. A neighbor-joining (NJ) tree was constructed and bootstrap resampling (1000 replicates) was conducted to assess the confidence in phylogenetic analysis by MEGA 6.0. The combination of ITS2 and *psbA*-*trnH* (ITS2 + *psbA*-*trnH*) was also evaluated by these methods.

## Results

### Efficiency of DNA extraction and PCR amplification

DNA was successfully extracted from all 75 samples. The PCR amplification success rates for both ITS2 and *psbA*-*trnH* were 100 %. All PCR products in correspondence to the ITS2 and *psbA*-*trnH* regions were successfully sequenced, and high-quality bidirectional sequences were obtained (Table 2).

**Table 2:** Characteristics of the DNA barcodes evaluated in this study

DNA region	ITS2	*psbA*-*trnH*	ITS2 + *psbA*-*trnH*
Number of individuals	86	75	75
Number of species	7	7	7
PCR/sequencing success (%)	100/100	100/100	100/100
Amplified sequence length (bp)	221–223	300–313	521–530
Aligned sequence length (bp)	227	320	547
Average GC content (%)	52.72	25.62	37.18
Variable sites	43	19	59
Haplotypes	23	23	28
Intra-specific distance range (mean)	0–0.0571 (0.0041)	0–0.0340 (0.0021)	0–0.0297 (0.0025)
Inter-specific distance range (mean)	0–0.1298 (0.0594)	0–0.0489 (0.0237)	0.0019–0.0708 (0.0363)

### Sequence and inter-/intra-specific variation analysis

The sequence characteristics are summarized in Tables 2 and 3. The average G-C contents of the ITS2 and *psbA*-*trnH* regions were 52.72 and 25.62 %, respectively. ITS2 sequences ranged from 221 to 223 bp with 43 variable sites; 23 haplotypes were identified, and four indels that were 1–2 bp in length within the aligned 227 bp. The *psbA*-*trnH* intergenic spacer region ranged from 300 to 313 bp and showed less variation, with only 19/320 variable sites among 23 haplotypes.

**Table 3:** Sequence information and intra/inter-specific genetic distance of ITS2, *psbA*-*trnH* and ITS2 + *psbA*-*trnH* regionss of *Hippophae* species

Species	ITS2				*psbA*-*trnH*				ITS2 + *psbA*-*trnH*			
	Length (bp)	GC content (%)	Intraspecific distance (mean)	Intrespecific distance (mean)	Length (bp)	GC content (%)	Intraspecific distance (mean)	Intrespecific distance (mean)	Length (bp)	GC content (%)	Intraspecific distance (mean)	Intrespecific distance (mean)
*H. rhamnoides*	223	52.4	0–0.0571 (0.0174)	0.0137–0.1190 (0.0644)	307	25.5	0–0.0340 (0.0142)	0–0.0447 (0.0212)	530	37	0–0.0297 (0.0127)	0.0019–0.0623 (0.0306)
*H. goniocarpa*	221	54.3	0	0.0137–0.0928 (0.0354)	307	25.4	0	0–0.0376 (0.0160)	528	37.5	0	0.0077–0.0478 (0.0236)
*H. litangensis*	221	52.5	0	0–0.1246 (0.0566)	303	25.1	0	0.0033–0.0411 (0.0173)	524	36.6	0	0.0038–0.0623 (0.0318)
*H. neurocarpa*	221	52.3	0–0.0091 (0.0031)	0–0.1298 (0.0587)	305	25.6	0	0–0.0341 (0.0172)	526	36.9	0–0.0038 (0.0013)	0.0038–0.0603 (0.0331)
*H. salicifolia*	223	52.5	0	0.0091–0.1142 (0.0474)	300	27	0	0.0135–0.0489 (0.0365)	523	37.9	0	0.0116–0.0708 (0.0398)
*H. gyantsensis*	221	52.4	0–0.0045 (0.0019)	0.0091–0.1198 (0.0487)	313	25.1	0–0.0032 (0.0008)	0.0135–0.0449 (0.0317)	534	36.3	0–0.0038 (0.0013)	0.0116–0.0685 (0.0377)
*H. tibetana*	223	59.4	0–0.0183 (0.0060)	0.0822–0.1298 (0.1050)	303	25.8	0	0.0133–0.0413 (0.0257)	526	40.1	0–0.0077 (0.0025)	0.0435–0.0708 (0.0575)

**Fig. 1:**
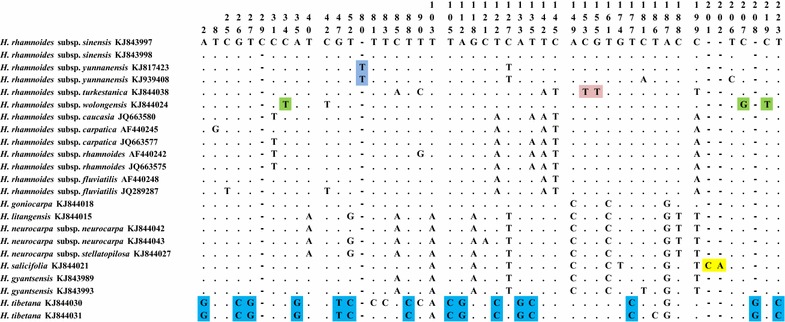
Variable sites and deletions for *Hippophae* species based on ITS2 sequences. The specific variable sites and deletions are *highlighted*

**Fig. 2:**
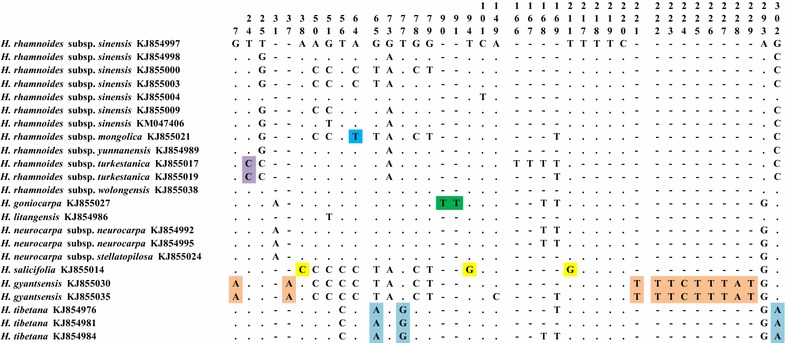
Variable sites and insertions for *Hippophae* species based on *psbA*-*trnH* sequences. The specific variable sites and deletions are *highlighted*

With these ITS2 sequences, both variable sites and deletions provided insight into the identification of *H. salifocilia*, *H. tibetana,* and three *H. rhamnoides* subspecies (Fig. 1). By comparing the sequences, all species except *H. salifocilia* have deletions from the sites 201–202; in *H. tibetana*, there were 15 variable sites from site 2 to site 223 which could be used for identification and discrimination from other species. Other important variable sites also provided useful information for species identification and discrimination, such as *H. rhamnoides* subsp. *yunnanensis* at site 80, *H. rhamnoides* subsp. *turkestanica* at site 153 and site 155, and *H. rhamnoides* subsp. *wolongensis* at site 34, site 207, and site 219. With *psbA*-*trnH* sequences, the variable sites and insertions enable the identification and differentiation of *H. goniocarpa, H. gyantsensis, H. salicifolia*, *H. tibetana,* and two *H. rhamnoides* subspecies (Fig. 2). When the sequences were compared, most species had no insertions except *H. goniocarpa*, which had insertions between site 90 and site 91, and *H. gyantsensis*, which had insertions at site 37 and from site 221 to site 229. Stable sequence variations, which provided useful information for species identification, were found in three species and two subspecies: *H. salicifolia* at site 38, site 94, and site 211; *H. gyantsensis* at site 7; *H. tibetana* at site 65, site 77, and site 302; *H. rhamnoides* subsp. *mongolica* at site 64; and *H. rhamnoides* subsp. *turkestanica* at site 24.

The intra- and inter-specific K2P genetic distances for ITS2, *psbA*-*trnH*, and ITS2 + *psbA*-*trnH* are listed in Table 2. In general, the mean inter-specific distances were higher than the mean intra-specific distances for the single-locus barcodes as well as the 2-locus barcode by the K2P model. ITS2 showed the highest intra- and inter-specific distances among the two DNA regions and the combination of the two regions, whereas the *psbA*-*trnH* exhibited the lowest intra- and inter-specific distances.

### Assessment of barcoding gaps

**Fig. 3:**
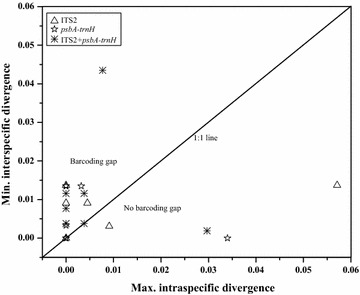
Barcoding gap between *Hippophae* species based on intra- and inter-specific distances. Minimum inter-specific K2P distance vs. maximum intra-specific K2P distance for ITS2, *psbA*-*trnH*, and ITS2 + *psbA*-*trnH*. Each data point represents a species, and each species located above the 1:1 line has a barcoding gap

Ideal barcode sequences should have a distinct inter-specific distance and relatively little intra-specific variation, and there need to be distinct differences between the sequences to form a spacer region, known as the “barcoding gap”. Figure 3 shows the minimum inter-specific K2P distances *vs.* maximum intra-specific distances, and the points that represented species distributed above the 1:1 line indicated that there were barcoding gaps for these species. With *psbA*-*trnH* and ITS2 + *psbA*-*trnH*, the species located in the area with no barcoding gap was *H. rhamnoides*. With the ITS2 region, there were two species, *H. rhamnoides* and *H. neurocarpa*, that had no barcoding gap. There were four points located on the 1:1 line, indicating that these species also had no barcoding gap. These four points included *H. litangensis* with ITS2, *H. goniocarpa* and *H. neurocarpa* with *psbA*-*trnH*, and *H. neurocarpa* with ITS2 + *psbA*-*trnH*.

### Neighbor-joining tree analysis

**Fig. 4:**
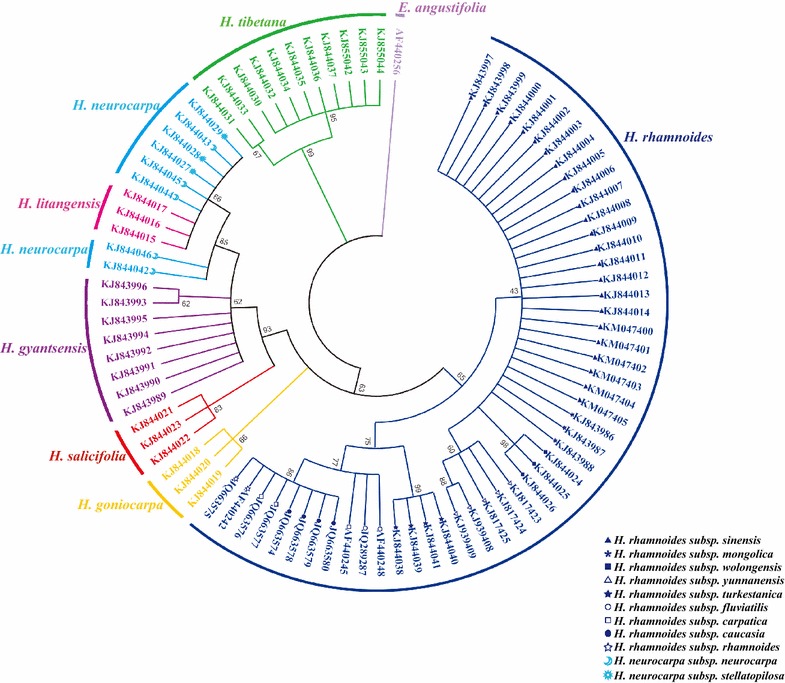
NJ tree of *Hippophae* constructed using ITS2. An *E. angustifolia* sequence downloaded from GenBank was included as an outgroup. The bootstrap scores (1000 replicates) are shown (≥50 %) for each branch. Each *color* represents one species

**Fig. 5:**
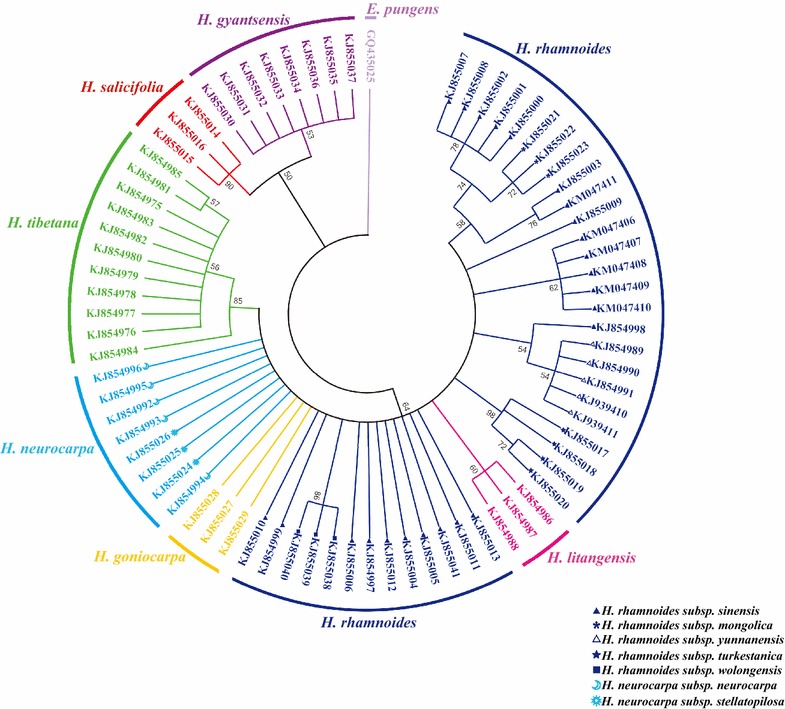
NJ tree of *Hippophae* constructed using *psbA*-*trnH*. An *E. pungens* sequence downloaded from GenBank was included as an outgroup. The bootstrap scores (1000 replicates) are shown (≥50 %) for each branch. Each *color* represents one species

**Fig. 6:**
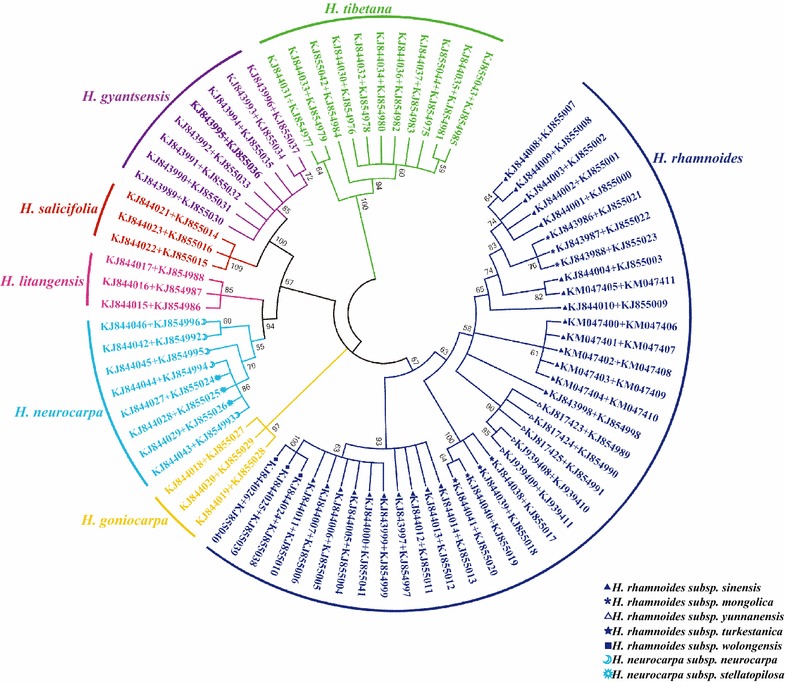
NJ tree of *Hippophae* constructed using ITS2 + *psbA*-*trnH*. The bootstrap scores (1000 replicates) are shown (≥50 %) for each branch. Each *color* represents one species

In this study, a phylogenetic tree was constructed by the NJ method, with 1000 bootstrap replicates for ITS2 (Fig. 4), *psbA*-*trnH* (Fig. 5), and ITS2 + *psbA*-*trnH* (Fig. 6) regions. Using ITS2 + *psbA*-*trnH* was the most effective for the species differentiation: all species were clearly identified, including the medicinal and non-medicinal *Hippophae* species. The ITS2 single-locus barcode was the second-most effective and differentiated five species: *H. rhamnoides*, *H. goniocarpa*, *H. salicifolia*, *H.gyantsensis*, and *H. tibetana*. The *psbA*-*trnH* region showed relatively poor performance with regard to species identification, as only four species were identified: *H. litangensis*, *H. salicifolia*, *H.gyantsensis*, and *H. tibetana*.

At the subspecies level, four subspecies were identified by *psbA*-*trnH* (*H. rhamnoides* ssp. *mongolica*, *H. rhamnoides* ssp. *yunnanensis*, *H. rhamnoides* ssp*. turkestanica*, and *H. rhamnoides* ssp. *wolongensis*), three subspecies with ITS2 (*H. rhamnoides* ssp. *yunnanensis*, *H. rhamnoides* ssp*. turkestanica*, and *H. rhamnoides* ssp. *wolongensis*), and four subspecies with ITS2 + *psbA*-*trnH* (*H. rhamnoides* ssp. *mongolica*, *H. rhamnoides* ssp. *yunnanensis*, *H. rhamnoides* ssp*. turkestanica*, and *H. rhamnoides* ssp. *wolongensis*). Consequently, the 2-locus barcode ITS2 + *psbA*-*trnH* showed the highest efficiency for identifying *Hippophae* at the species and subspecies level. The single-locus barcode *psbA*-*trnH* was also suitable for identifying *H. rhamnoides* subspecies.

## Discussion

The morphological similarities of *Hippophae* species caused a high chance of misidentification and misuse. Raw *Hippophae* products are often sold in dried and powdered forms, making morphological identification infeasible.

DNA barcoding is an important supplement and validation of conventional morphological identification [23]. In the present study, medicinal and non-medicinal *Hippophae* species were identified by DNA barcoding after a preliminary morphological identification, and remarkable *Hippophae* variation at the species level was shown. The genomic DNA could be extracted from dried fruits with both ITS2 and *psbA*-*trnH* with 100 % amplification and sequencing efficiencies. Two single-locus barcodes, ITS2 and *psbA*-*trnH*, as well as their combination were evaluated and validated. All *Hippophae* species were successfully identified by DNA barcoding, and four *H. rhamnoides* subspecies were also differentiated. The information obtained from the variable sequence sites and deletions/insertions facilitated the identification of *Hippophae* species; *H. salicifolia*, *H. tibetana*, and three *H. rhamnoides* subspecies were identified by ITS2 sequences, whereas *H. goniocarpa, H. salicifolia, H. gyantsensis*, *H. tibetana*, and two *H. rhamnoides* subspecies were identified by *psbA*-*trnH* sequences.

A relatively high value was observed for ITS2 + *psbA*-*trnH* with regard to the barcoding gap analysis: one species was located under the 1:1 line, and one species was located on the 1:1 line. However, three species had no barcoding gap for each of the single-locus barcodes: *H. rhamnoides*, *H. litangensis*, and *H. neurocarpa* for ITS2 barcode; *H. rhamnoides*, *H. goniocarpa*, and *H. neurocarpa* for *psbA*-*trnH* barcode. The identification efficiency of single-locus and combined barcodes by the NJ tree method showed that ITS2 + *psbA*-*trnH* was the most suitable barcode, with all seven species as well as four *H. rhamnoides* subspecies clearly identified. None of the selected barcodes were suitable for *H. neurocarpa* subspecies identification. Although it was hard to identify all *H. rhamnoides* and *H. neurocarpa* subspecies by ITS2, *psbA*-*trnH*, and ITS2 + *psbA*-*trnH*, the medicinal species were successfully distinguished from non-medicinal *Hippophae* species. While *H. rhamnoides* is the original medicinal plant according to Chinese Pharmacopeia, *H. neurocarpa*, *H. gyantsensis*, and *H. tibetana* are used in the Tibetan medicine [14, 15, 17, 18]. Thus, all native *Hippophae* species were identified by DNA barcode and the accurate and standard sequence information was gained. This information would be applicable to commercial products alignment and authenticate *Hippophae* species origins in the future.

There have been debates over whether *H. litangensis* was a subspecies of *H. goniocarpa* and whether *H. rhamnoides* subsp. *wolongensis* was a distinct species [3, 4, 42]. In our study, we considered *H. litangensis* and *H. goniocarpa* as two separate species, and the results demonstrated that they could be identified separately at the species level; *H. rhamnoides* subsp. *wolongensis* was a subspecies of *H. rhamnoides* based on the K2P genetic distance, NJ tree, and identification efficiency results.

## Conclusion

The combination of the two loci, ITS2 + *psbA*-*trnH* is applicable to the identification of medicinal and non-medicinal *Hippophae* species.

## Abbreviations

ITS2: internal transcribed spacer 2
K2P: Kimura 2-parameter
NJ tree: neighbor-joining tree
CM: Chinese medicine
CBOL: Consortium for the Barcode of Life
